# Efficacy and safety of first-line pemetrexed plus carboplatin followed by single-agent pemetrexed maintenance in elderly Chinese patients with non-squamous non-small-cell lung cancer

**DOI:** 10.18632/oncotarget.21186

**Published:** 2017-09-23

**Authors:** Xinmin Zhao, Hui Yu, Jing Zhao, Xianghua Wu, Si Sun, Zhiguo Luo, Huijie Wang, Jie Qiao, Jianhua Chang, Jialei Wang

**Affiliations:** ^1^ Department of Medical Oncology, Fudan University Shanghai Cancer Center, Department of Oncology, Shanghai Medical College, Fudan University, Shanghai 200032, China; ^2^ Department of Medical Oncology, Shanghai Pulmonary Hospital Affiliated to Tongji University, Tongji University Medical School Cancer Institute, Shanghai 200433, China

**Keywords:** non-small-cell lung cancer, pemetrexed, carboplatin, elderly, maintenance therapy

## Abstract

Chemotherapy with pemetrexed plus carboplatin followed by pemetrexed maintenance therapy is a first-line regimen for patients with advanced non-squamous non-small-cell lung cancer. This phase II clinical study investigated the efficacy and safety of this regimen in older patients (aged ≥65 years) with advanced non-squamous non-small-cell lung cancer. All patients received 4 courses of induction therapy with pemetrexed (500 mg/m^2^) combined with carboplatin once every 3 weeks. If patients had stable disease or achieved a complete or partial tumor response after 4 courses of pemetrexed + carboplatin therapy, maintenance treatment with pemetrexed monotherapy was administered until either disease progression or intolerable toxicity occurred. The primary endpoint was progression-free survival, while secondary endpoints were the objective response rate, overall survival, and tolerability. A total of 105 elderly patients (median age, 71 years) with advanced lung adenocarcinoma were enrolled in the trial. The ORR with induction therapy was 36.2% and the disease control rate was 70.5%. Sixty-two patients (59.0%) subsequently received pemetrexed maintenance therapy. The median progression-free survival for all patients was 8.23 months (95% CI 5.85-10.62 months) and the median overall survival was 22.6 months (95% CI 20.09-25.11 months). Grade 3 or greater toxicities included neutropenia (15.3%), thrombocytopenia (9.5%), anemia (8.6%), leukopenia (4.8%), nausea (1.0%), vomiting (1.0%), and fatigue (1.0%). No treatment-related deaths occurred. These results indicate that pemetrexed combined with carboplatin therapy maintained by single-agent pemetrexed treatment of elderly patients with advanced non-squamous non-small-cell lung cancer was effective and tolerable.

ClinicalTrials.gov identifier: NCT01860508.

## INTRODUCTION

Lung cancer is the most common malignancy, and in 2015 it was the most common cause of cancer-related deaths in both men and women. About 85% of lung cancers are non-small-cell lung cancer (NSCLC), and in 80% of patients, the initial diagnosis is made late [1]. In patients with advanced, non-squamous NSCLC, first-line chemotherapy with pemetrexed plus a platinum agent followed by pemetrexed maintenance therapy has become the standard chemotherapy regimen. This regimen has been found to improve overall survival (OS) by 2.9 months versus placebo, and reduce the risk of disease progression by 38% and death by 22% [2, 3].

In the elderly (the WHO definition of which is age ≥65 years), NSCLC occurrence and death rates are increased relative to younger patients [4]. NSCLC is often seen in the elderly, and a median age of onset of 70 years has been reported [5]. Treatment of lung cancer in elderly patients is more complicated than in younger patients because the elderly often have other coexisting disorders such as diabetes, chronic obstructive pulmonary disease, cardiovascular and cerebrovascular diseases, and osteoporosis. Secondly, due to memory loss, medical diagnosis and treatment of this population is often late [6]. Thirdly, bone marrow reserve capacity and liver metabolic capacity are also reduced in elderly patients in addition to diminished heart and kidney function [7]. Moreover, elderly patients require more medication and are more prone to water and electrolyte disturbances.

Although chemotherapy cannot sometimes be administered to elderly patients, the long-term survival of 70-year-old patients, both male and female, is expected to be 10 to 15 years, and even 80-year-old patients can be expected to survive for 7-9 years [6, 8, 9]. Despite the occurrence of complications and coexisting medical disorders, there are often opportunities for ongoing chemotherapy in elderly individuals with a good Eastern Cooperative Oncology Group performance status (ECOG PS). In choosing treatments for elderly patients, agents with as little cardiovascular and renal toxicity as possible, and agents without other major adverse effects or prominent drug interaction potentials are preferred [10]. Theoretically, pemetrexed combined with carboplatin has the potential to be an ideal regimen for elderly patients with advanced non-squamous NSCLC. No significant differences in the pharmacokinetics of pemetrexed have been noted in patients aged 26 to 80 years [11–14], and an analysis of an elderly subgroup (>70 years of age) in the PARAMOUNT study showed that maintenance pemetrexed treatment following induction therapy with pemetrexed and cisplatin was effective with manageable toxicities [2, 3].

Based on these findings, we initiated a single-arm, open-label clinical study to evaluate the efficacy and safety of pemetrexed in combination with carboplatin followed by single-agent pemetrexed maintenance therapy in elderly Chinese patients (aged ≥65 years) with advanced non-squamous NSCLC.

## RESULTS

### Patient characteristics

Between March 2013 and December 2015, a total of 105 patients were enrolled in the study. Their characteristics are shown in Table 1. The patients’ median age was 71 years, 58.1% were male, 51.4% had an ECOG PS of 0, 89.5% had stage IV NSCLC, 37.1% had an *EGFR* exon 19 mutation, 15.2% had an *EGFR* exon 21 mutation, none had an *ALK* translocation, 43.8% had bone metastases, 58.1% were never smokers, and 40% had coexisting morbidities.

**Table 1:** Characteristics of the 105 patients

Patient characteristics	n^a^	%
Age, years (median, range)	71 (65-81)	
Sex:		
Male	61	58.1
Female	44	41.9
ECOG performance status:		
0	54	51.4
1	51	48.6
Stage:		
IIIB	11	10.5
IV	94	89.5
*EGFR* mutation:		
Exon 18	1	0.9
Exon 19	39	37.1
Exon 20	3	2.9
Exon 21	16	15.2
Wild-type	40	38.1
Unknown	6	5.8
*ALK* translocation:		
Positive	0	0
Negative	88	83.8
Unknown	17	16.2
Metastatic sites:		
Supraclavicular lymph nodes	18	17.1
Bone	46	43.8
Lung	32	30.5
Liver	10	9.5
Brain	13	12.4
Effusion:		
Pleural effusion	35	33.3
Pericardial effusion	10	9.5
Adrenal gland	2	1.9
Other	4	3.7
Metastases:		
1-3	96	91.4
>3	9	8.6
Smoking history:		
Never	61	58.1
Current/former	44	41.9
Coexisting morbidities:		
None	63	60.0
Hypertension	26	24.8
Diabetes	7	6.7
Other	9	8.6
Weight loss >5kg/0.5 year:		
No	101	96.2
Yes	4	3.8

^a^ Unless otherwise specified.

Abbreviations: *ALK*, anaplastic lymphoma kinase; ECOG, Eastern Cooperative Oncology Group; *EGFR*, epidermal growth factor receptor.

### Efficacy

**Figure 1:**
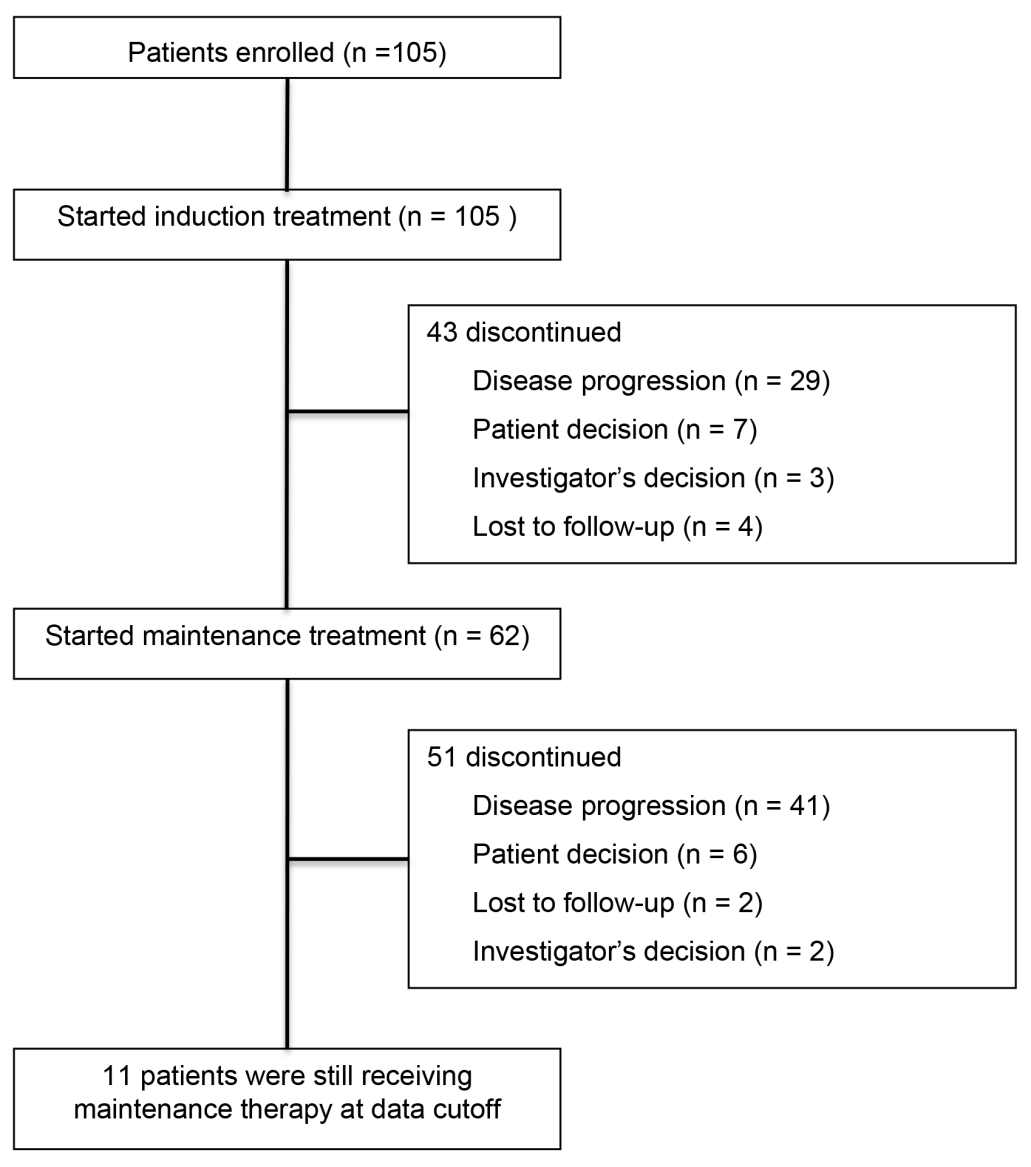
Study profile showing patient registration and numbers of patients who received induction treatment and maintenance therapy

**Figure 2:**
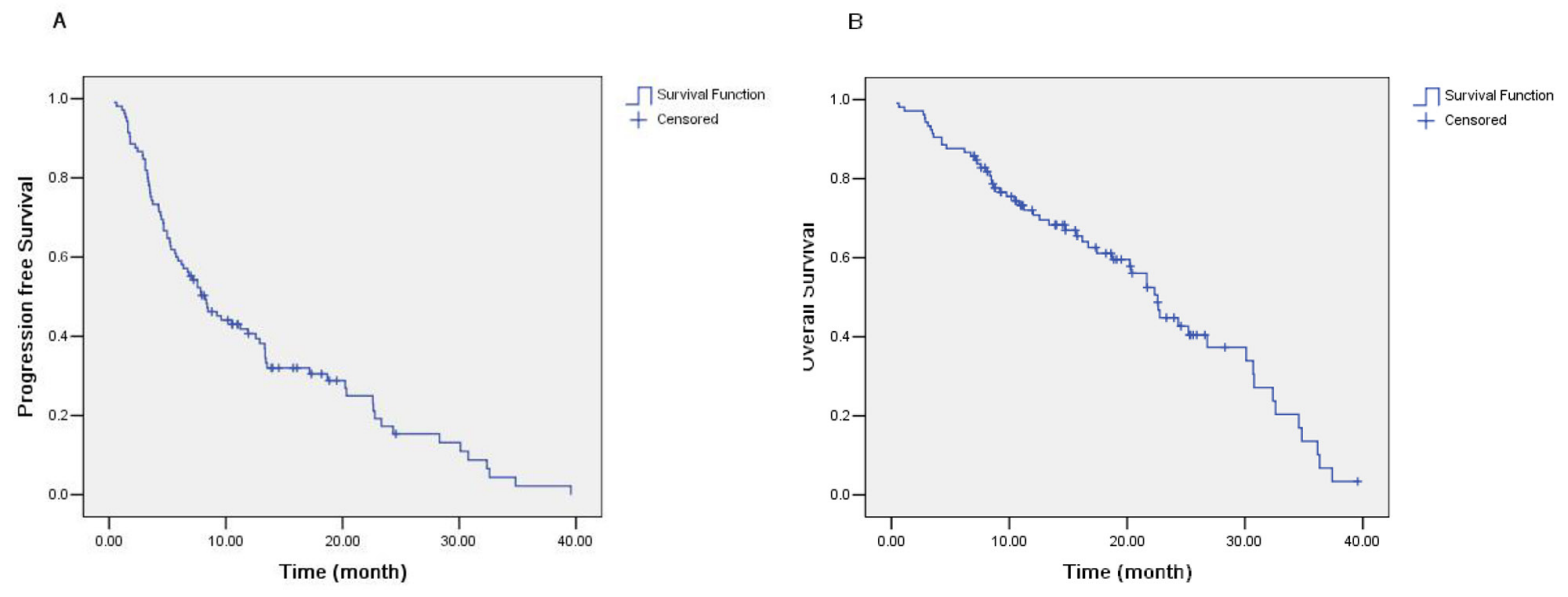
**(A)** Progression-free survival (PFS) of all patients (median, 8.23 months; 95% CI 5.85-10.62 months). **(B)** Overall survival (OS) of all patients (median, 22.60 months; 95% CI 20.09-25.11 months).

The tumor responses obtained with the induction therapy and maintenance therapy regimens are shown in Table 2. The median follow-up time was 14.0 months (range, 1.1-39.7 months) and 44.8% of the patients were still alive at the end of the study. With the induction regimen (pemetrexed + carboplatin), 36.2% (38/105) of patients achieved a partial response (PR) and 34.3% (36/105) had stable disease (SD), giving a disease control rate (DCR) of 70.5%. However, 2 PR patients and 10 SD patients did not receive maintenance therapy (Figure 1). Thus, a total of 62 patients (59%) received maintenance therapy; 1.6% (1/62) of these patients achieved a new PR and 72.6% (45/62) had SD, but 25.8% (16/62) experienced disease progression. The median progression-free survival (PFS) for all patients was 8.23 months (95% CI 5.85-10.62 months), and the median overall survival (OS) was 22.60 months (95% CI 20.09-25.11 months) (Figures 2A and 2B). Among the 56 patients with *EGFR*-sensitive mutations, the median PFS was 10.5 months (95% CI 3.3-17.7 months), and the median OS was 30.8 months (95% CI 29.5-32.0 months).

**Table 2:** Tumor responses to the initial induction therapy and maintenance therapy regimens

	n	%
**Responses to induction therapy (n = 105):**		
CR	0	0
PR	38	36.2
SD	36	34.3
PD	29	27.6
NE	2	1.9
**Responses to maintenance therapy (n = 62; 59.0%):**		
CR	0	0
PR	1	1.6
SD	45	72.6
PD	16	25.8
NE	0	0

Abbreviations: CR, complete response; NE, not evaluable; PD, progressive disease; PR, partial response; SD, stable disease.

PFS and OS data according to sex, ECOG PS, age, smoking history, *EGFR* mutation status, and the tumor response are shown in Tables 3 and 4. Univariate analysis of the PFS data showed that female patients and those with an ECOG PS of 0, an *EGFR*-sensitive mutation, no smoking history, and a tumor response had a significantly longer median PFS (P = 0.037, 0.01, 0.035, 0.007, and 0.000, respectively). Multivariate analysis showed that an ECOG PS status of 0 and no smoking history were associated with a significantly longer PFS (Table 3). Similarly, univariate analysis of OS data showed that female patients and those with an ECOG PS of 0, no smoking history, an *EGFR*-sensitive mutation, and a tumor response had a significantly longer median OS (P = 0.026, 0.000, 0.011, 0.000, and 0.000, respectively). Multivariate analysis indicated that an ECOG PS status of 0 and female sex were associated with a significantly longer OS (Table 4).

**Table 3:** PFS according to sex, age, ECOG performance status, *EGFR* mutation status, smoking history, and the tumor response

	Median PFS, months (95% CI)	Univariate analysis: P-value	Multivariate analysis	
			HR (95% CI)	P-value
Sex:		0.037		
Male	7.1 (3.5-10.8)			
Female	9.6 (3.1-16.1)			
Age, years:		0.382		
<70	7.5 (5.0-10.1)			
≥70	9.2 (3.4-15.0)			
ECOG PS:		0.01	2.06 (1.31-3.23)	0.002
0	13.3 (9.2-17.4)			
1	5.2 (2.2-8.2)			
*EGFR* mutation status:		0.035		
Wild-type	5.3 (2.2-8.3)			
Sensitive mutation	10.5 (3.3-17.7)			
Unknown	8.2 (5.1-11.4)			
Smoking history:		0.007	1.75 (1.12-2.75)	0.014
Never	11.9 (6.1-17.7)			
Current/former	5.7 (3.1-8.4)			
Tumor response:		0.000		
PR	29.7 (24.9-34.5)			
SD	6.7 (4.0-9.5)			
PD	3.1 (1.9-4.3)			
NE	3.7 (1.2-10.4)			

Abbreviations: ECOG, Eastern Cooperative Oncology Group; *EGFR*, epidermal growth factor receptor; HR, hazard ratio; NE, not evaluable; PD; progressive disease; PFS, progression-free survival; PR, partial response; PS, performance status; SD, stable disease.

**Table 4:** OS according to sex, age, ECOG performance status, *EGFR* mutation status, smoking history, and the tumor response

	Median OS, months (95% CI)	Univariate analysis: P-value	Multivariate analysis	
			HR (95% CI)	P-value
Sex:		0.026	2.35 (1.32-4.19)	0.004
Male	16.7 (10.9-22.5)			
Female	30.8 (20.8-40.7)			
Age, years		0.948		
<70	25.2 (20.3-30.1)			
≥70	21.7 (17.9-25.4)			
ECOG PS:		0.000	5.01 (2.68-9.34)	0.000
0	26.8 (16.5-37.1)			
1	10.4 (6.5-14.3)			
*EGFR* mutation status:		0.000		
Wild-type	10.4 (7.0-13.9)			
Sensitive mutation	30.8 (29.5-32.0)			
Unknown	17.4 (3.3-31.6)			
Smoking history:		0.011		
Never	24.3 (16.3-32.3)			
Current/former	13.4 (7.0-19.8)			
Tumor response:		0.000		
PR	30.0 (21.9-38.2)			
SD	21.7 (15.0-28.3)			
PD	12.0 (4.4-19.6)			
NE	3.8 (1.4-10.4)			

Abbreviations: ECOG, Eastern Cooperative Oncology Group; *EGFR*, epidermal growth factor receptor; HR, hazard ratio; NE, not evaluable; PD; progressive disease; PFS, progression-free survival; PR, partial response; PS, performance status; SD, stable disease.

### Additional therapy after first-line chemotherapy

A total of 56 patients received sequential treatment. Thirty-five patients received the study chemotherapy regimen first followed by tyrosine kinase inhibitor (TKI) treatment, while 21 patients received docetaxel monotherapy as second-line chemotherapy.

### Tolerability

The principal adverse events observed in the 105 enrolled patients are shown in Table 5. Leukopenia of all grades of severity was noted in 55 patients (52.4%), anemia in 44 (42.0%), neutropenia in 49 (48.7%), and thrombocytopenia in 27 (25.7%). Non-hematologic toxicity of all grades of severity included nausea in 51 patients (48.6%), vomiting in 40 (38.2%), rash in 18 (17.2%), fatigue in 14 (13.4%), and liver enzyme elevations in 10 (9.6%). Grade 3 or 4 adverse events included leukopenia in 5 patients (4.8%), neutropenia in 16 (15.3%), thrombocytopenia in 10 (9.5%), anemia in 9 (8.6%), nausea in 1 (1.0%), vomiting in 1 (1.0%), and fatigue 1 (1.0%). There were no treatment-related deaths.

**Table 5:** Adverse events occurring in the 105 patients

Toxicity	Total events (n; %)	Grade <3 (n; %)	Grade ≥3 (n; %)
Leukopenia	55 (52.4)	50 (47.6)	5 (4.8)
Neutropenia	49 (48.7)	33 (31.4)	16 (15.3)
Anemia	44 (42.0)	35 (33.4)	9 (8.6)
Thrombocytopenia	27 (25.7)	17 (16.2)	10 (9.5)
Increased AST	10 (9.6)	10 (9.6)	0 (0)
Increased ALT	10 (9.6)	10 (9.6)	0 (0)
Increased TBI	5 (4.8)	5 (4.8)	0 (0)
Nausea	51 (48.6)	50 (47.6)	1 (1.0)
Vomiting	40 (38.2)	39 (37.2)	1 (1.0)
Fatigue	14 (13.4)	13 (12.4)	1 (1.0)
Rash	18 (17.2)	18 (17.2)	0 (0)

Abbreviations: ALT, alanine aminotransferase; AST, aspartate aminotransferase; TBI, total bilirubin.

## DISCUSSION

This single-arm, open-label clinical study evaluated the efficacy and safety of pemetrexed in combination with carboplatin as first-line therapy followed by single-agent pemetrexed maintenance therapy in elderly Chinese patients (≥65 years of age) with non-squamous NSCLC. This regimen showed good efficacy and tolerability. OS, PFS, and objective response rate (ORR) values and adverse events were comparable to those reported in previous studies [2, 3, 15].

A recent phase II study by Tamiya et al. [15] found that pemetrexed (500 mg/m^2^) plus carboplatin (AUC = 5) followed by maintenance pemetrexed therapy in 34 elderly patients with stage IIIB or IV NSCLC resulted in a 1-year survival rate of 58.0%, and an ORR and DCR of 41.2% and 85.3%, respectively. The median PFS was 5.7 months (95% CI 3.9-8.9 months), and the median OS was 20.5 months. As in our study, the chemotherapy regimen was reasonably well tolerated. Other studies have shown that a platinum-based induction regimen followed by maintenance therapy is superior to single-agent chemotherapy [16].

In our study, 59% of the patients received the maintenance therapy regimen. This was consistent with the PARAMOUNT study (57.4%) [2, 3], the study of Tamiya et al. (58.8%) [15], and the JACAL study (57.4%) [16]. As with our study, the study of Tamiya et al. [15] was conducted in elderly patients who received pemetrexed + carboplatin as induction therapy, while the PARAMOUNT and JACAL studies both targeted the general population and only retrospectively analyzed older patients. Also, the PARAMOUNT study investigated pemetrexed combined with cisplatin, while our study and the JACAL [16] and Tamiya et al. [15] studies investigated pemetrexed combined with carboplatin for induction therapy. The study population in our study and the trial design were similar to that of the study of Tamiya et al. [15], and the ORR and DCR were comparable to that of the PARAMOUNT study [2, 3].

Although the statistical power of our study was not very high, the PFS and OS values achieved were relatively long, 8.23 months and 22.6 months, respectively, and similar to those obtained in other studies. The possible reasons for this are: (1) while the patients in our study were elderly, their ECOG PS was good (all had a PS <2 and about half had a PS of 0); (2) the number of patients with greater than 3 metastases in our study population was less than 10%; (3) the nutritional status of our patients was good (only 3.8% had weight loss of 5 kg over 6 months); and (4) we gave standard second-line treatment to patients. Patients with sensitive *EGFR* mutations received EGFR-TKIs after the failure of maintenance therapy. Docetaxel was administered to patients with *EGFR* wild-type or to patients with *EGFR* mutations whose disease had progressed after EGFR-TKI therapy.

Interestingly, in our study, we found that *EGFR*-mutated patients exhibited superior efficacy, as did female patients and those with an ECOG PS of 0 and no smoking history. These patient groups achieved a longer PFS and OS with the study chemotherapy regimen, and follow-up targeted therapy also contributed to their longer survival.

The results of the IFCT-0501 phase III study [17], which was the first clinical study to fully investigate elderly patients with advanced NSCLC, demonstrated that weekly paclitaxel plus carboplatin therapy was superior to gemcitabine or vinorelbine monotherapy. PFS and OS were longer with the carboplatin-based doublet chemotherapy than with the monotherapy regimens (PFS: 6.0 months vs 2.8 months, respectively; OS: 10.3 months vs 6.2 months, respectively). In addition, the 1-year survival rate was increased from 25.4% with the monotherapy regimens to 44.5% with the carboplatin-based doublet regimen, indicating that the latter can provide survival benefits. In most cases, toxicity was controllable with the doublet regimen, even in the subgroup with a poor prognosis [17]. In another multicenter, phase III, randomized trial, in which patients with advanced non-squamous NSCLC (ECOG PS = 2) were randomly assigned to receive single-agent pemetrexed or pemetrexed + carboplatin therapy, the median PFS was 2.8 months and 5.8 months, respectively (hazard ratio [HR], 0.46; 95% CI 0.35 to 0.63; P < 0.001), and the median OS was 5.3 months and 9.3 months, respectively (HR, 0.62; 95% CI 0.46 to 0.83; P = 0.001) [18]. In this study, approximately 35% of the patients enrolled were aged ≥70 years, and in the elderly subsets (n = 36 and n = 38, respectively), the median OS with single-agent pemetrexed and pemetrexed + carboplatin therapy was 5.3 months and 9.9 months, respectively (HR, 0.49; 95% CI 0.29 to 0.82; P = 0.006). Thus, the combination of pemetrexed and carboplatin significantly improved survival in patients with advanced NSCLC with an ECOG PS of 2, even in elderly patients. Our results were better than these 2 studies. Possible explanations for this are: (1) our study included maintenance therapy, while the other 2 studies only gave induction therapy without maintenance therapy; (2) the ECOG PS of the patients in our study was good, with all having a PS <2 and about half having a PS of 0; (3) the number of patients with metastases in our study was relatively less; and (4) the age of patients in our study was relatively less than that of patients in the IFCT 0501 study [17].

The incidences of hematological and non-hematologic adverse events in our study were comparable to those reported in other studies, and whether they were greater than grade 3 in severity or not, they were manageable and able to be controlled. In the phase II study of Tamiya et al. [15] in Japanese patients, grade 3 hematologic toxicity was observed in 56% of patients and interstitial lung disease (ILD) occurred in 2.9%. Our study enrolled patients older than 65 years of age, while Tamiya et al. [15] enrolled patients ≥75 years of age. 51.4% of the patients in our study had an ECOG PS of 0 and all had a PS less than 2, but only 38% of the patients in the study of Tamiya et al. had a PS of 0, which could explain why tolerance in our study was much better than that observed in the Japanese patients [15]. Cardiac and renal toxicity were evaluated in all enrolled patients, and were not observed during the study. The occurrence of hematologic and non-hematologic adverse events in our study were less than those reported in the IFCT 0501 study [17], especially neuropathy, hair loss, and grade 3/4 hematological toxicities, which suggests that pemetrexed in combination with carboplatin was safer than weekly paclitaxel plus carboplatin and more suitable for elderly patients.

There are some limitations of our study. Firstly, as the study was a single-arm, open-label clinical trial, some bias in patient selection may have been present. Secondly, because of the early start time of the study, our study included patients with *EGFR* mutations and *EGFR* wild-type. Patients with *EGFR* mutations and *EGFR* wild-type have different prognoses, which may have had some influence on survival. Thirdly, the sequential treatments may have affected the results.

In conclusion, the findings of this study indicate that pemetrexed plus carboplatin followed by single-agent pemetrexed maintenance therapy was effective and safe in elderly Chinese patients (aged ≥65 years) with advanced or metastatic non-squamous NSCLC. This regimen may be considered first-line chemotherapy in this population.

## MATERIALS AND METHODS

### Patients

Patients were eligible for the study if they were aged at least 65 years, had an ECOG PS score of 0-1, a histologically- or cytologically-confirmed diagnosis of non-squamous NSCLC, and clinical stage IIIB disease not amenable to multimodality treatment or stage IV disease. Patients were required to have received no prior systemic chemotherapy for advanced, recurrent or metastatic NSCLC, have a life expectancy of at least 3 months, measurable lesions according to the Response Evaluation Criteria in Solid Tumors (RECIST version 1.1) guidelines, and adequate hematological, hepatic, and renal function (including a glomerular filtration rate ≥45 mL/min). Patients with previous brain metastases were eligible if they were asymptomatic. Those with neurological symptoms or signs due to brain metastases were required to undergo brain radiotherapy and be clinically stable. If radiation treatment had been administered, it needed to be completed at least 14 days prior to enrollment.

Exclusion criteria included active malignancies within the past 5 years (with the exception of *in situ* carcinoma of the cervix or basal cell carcinoma of the skin), uncontrolled pleural effusion, uncontrolled diabetes, or any clinically significant concomitant disease or condition that could interfere with the pemetrexed premedication.

The protocol for the trial was approved by the institutional review board of Fudan University, Shanghai Cancer Center, and the study adhered to the ethical principles set out in the Declaration of Helsinki. All enrolled patients provided written informed consent. The study was registered with ClinicalTrial.gov (identifier: NCT01860508).

### Treatment

All patients received pemetrexed 500 mg/m^2^ by a 10-minute intravenous infusion followed by carboplatin (area under the concentration-time curve [AUC] 5 mg/mL∙min) intravenously over 120 minutes on day 1 of each 3-week cycle, for a maximum of 4 cycles. Following the completion of 4 cycles of pemetrexed + carboplatin induction treatment, single-agent pemetrexed maintenance therapy was administered if the patient had achieved a complete response, partial response, or stable disease, and was continued until progressive disease or intolerant toxicity was observed. All patients received premedication with dexamethasone, vitamin B_12_, and folic acid according to the pemetrexed dosage recommendations in the datasheet.

The dosages of pemetrexed and carboplatin were modified if myelosuppression or hepatic dysfunction occurred. Dosage modifications were based on the worst toxicity observed during the previous treatment cycle. Treatment was delayed if hematologic recovery was inadequate (absolute neutrophil count <2.0 × 10^9^/L or a platelet count <80 × 10^12^/L) by day 21. For grade 2 or higher alanine aminotransferase (ALT) or aspartate aminotransferase (AST) elevations on day 21, the next cycle of treatment was delayed until the ALT/AST levels had recovered to grade 1 or to the upper limit of normal (ULN). If toxicity had not recovered within 42 days, the patient was excluded from the study. For febrile neutropenia or grade 4 neutropenia during the previous treatment cycle, the pemetrexed dosage was reduced by 25% and carboplatin dosage from AUC = 5 to AUC = 4 in the subsequent cycle.

### Evaluation of response and toxicity

All patients underwent comprehensive baseline assessments including a detailed medical history, a physical examination and determination of their performance status, clinical laboratory tests, and imaging studies. Laboratory evaluations included a routine blood count, urinalysis, fecal occult blood test, and renal and liver function tests. All patients underwent a pretreatment 12-lead electrocardiogram. Radiographic evaluation included computed tomography (CT) of the chest and upper abdomen, brain magnetic resonance imaging (MRI), and a bone scan. Complete blood counts were performed at least once a week during chemotherapy.

The serum chemistry profile and liver and renal function tests were monitored before each cycle of chemotherapy. The toxicity evaluation was based on the Common Terminology Criteria for Adverse Events, version 3.0 (NCI-CTCAE 3.0 version).

Clinical responses were evaluated according to RECIST (version 1.1) guidelines via CT scans performed every 2 cycles. Assessment of the tumor response during the maintenance therapy phase used the radiological evaluation before the first maintenance dosage as the baseline measurement. After discontinuation of treatment, patients were followed-up every 2 months and sequential therapy, progressive disease, and survival were recorded.

### Statistical analysis

The primary study endpoint was progression-free survival, while secondary endpoints were the tumor response, overall survival, and tolerability. Progression-free survival was defined as the time from enrollment to the date of confirmation of progressive disease or the date of death from any cause. Overall survival was defined as the time from enrollment until death from any cause. Patients whose survival or disease progression was unknown at the end of study were censored at the date of the last contact.

In the phase III PARAMOUNT study [2, 3], the median progression-free survival in the pemetrexed maintenance therapy group was 4.1 months (95% CI 3.2-4.6 months) as compared with 2.8 months (95% CI 2.6-3.6 months) in the placebo group. This study was based on the exponential distribution of the survival time, λ = -ln (0.5) / (2.8/12) in the placebo group and -ln (0.5) / (4/12) in the study group. The required number of patients for a 2-sided α-value of 0.05 and a β-value (power) of 0.8 was estimated at 94 for a follow-up time of 3 years. Therefore, for our study, considering that there may be about a 10% withdrawal or loss to follow-up rate, the target patient population was estimated to be 104 patients.

The main efficacy analysis was conducted on the full analysis set, which was determined after omission of ineligible patients. Kaplan-Meier plots were used to calculate PFS and OS, and the median and 95% confidence interval (CI) values were determined. Statistical significance was defined as P < 0.05. SPSS^®^ 13.0 (SPSS Inc., Cary, NC, USA) was used for all statistical analyses. The data lock date was June 29, 2016.

The distribution of the best overall response was summarized in patients with target lesions, and incidences of adverse events were recorded.
